# Effect of acupuncture depth on muscle pain

**DOI:** 10.1186/1749-8546-6-24

**Published:** 2011-06-22

**Authors:** Kazunori Itoh, Yoichi Minakawa, Hiroshi Kitakoji

**Affiliations:** 1 Department of Clinical Acupuncture and Moxibustion, Meiji University of Integrative Medicine, Hiyoshi-cho, Nantan, Kyoto 629-0392, Japan

## Abstract

**Background:**

While evidence supports efficacy of acupuncture and/or dry needling in treating musculoskeletal pain, it is unclear which needling method is most effective. This study aims to determine the effects of depth of needle penetration on muscle pain.

**Methods:**

A total of 22 healthy volunteers performed repeated eccentric contractions to induce muscle soreness in their extensor digital muscle. Subjects were assigned randomly to four groups, namely control group, skin group (depth of 3 mm: the extensor digital muscle), muscle group (depth of 10 mm: the extensor digital muscle) and non-segmental group (depth of 10 mm: the anterior tibial muscle). Pressure pain threshold and electrical pain threshold of the skin, fascia and muscle were measured at a point 20 mm distal to the maximum tender point on the second day after the exercise.

**Results:**

Pressure pain thresholds of skin group (depth of 3 mm: the extensor digital muscle) and muscle group (depth of 10 mm: the extensor digital muscle) were significantly higher than the control group, whereas the electrical pain threshold at fascia of muscle group (depth of 10 mm: the extensor digital muscle) was a significantly higher than control group; however, there was no significant difference between the control and other groups.

**Conclusion:**

The present study shows that acupuncture stimulation of muscle increases the PPT and EPT of fascia. The depth of needle penetration is important for the relief of muscle pain.

## Background

Recent clinical investigations on the myofascial pain syndrome and fibromyalgia focused on the existence of tender point and/or trigger point, with some literature emphasizing the importance of the tender and/or trigger points as diagnostic points [1-5]. Moreover these points have also been demonstrated as treatment points [6-9]. Sensitivity of tenderness and the number of tender points, being closely related to symptom severity can be used to evaluate the effectiveness of a certain treatment [1-5].

Acupuncture treatment includes tender or trigger points known as *Ah-Shi *or 'Oh-Yes' for treatments of myofascial pain syndrome and fibromyalgia. Studies have shown that application of acupuncture at these points improves symptoms in these disorders [10-12]. Needling points are most commonly chosen according to the anatomical structure or tenderness at the points, whereas the depth of needle penetration is determined with the consideration of the patient's sensation known as *deqi *and/or resistance felt by the practitioner [13]. Previously we have demonstrated that acupuncture with deeper insertion at the segmental muscle was more effective than that with shallow insertion in patients with chronic low back pain [14]. However, the required depth and site of needle penetration have not been determined.

In general, insertion of needles on the affected muscles affected sensitized nociceptors whereas skin insertion did not [15]. Muscle containing nociceptors such as polymodal-type receptor was demonstrated to be sensitized by various factors [15-17]. Sensitized polymodal-type receptors in the muscle lesion caused muscle pain [18] which was found mostly within the connective tissues of the muscle [19]. In particular, the fascia and/or muscle were the most sensitive deep tissues [16]. Most thin afferent fibers in muscle innervate polymodal-type receptors. Acupuncture stimulation at the affected muscle in myofascial pain could easily activate these receptors and consequently increase pain thresholds *via *internal analgesic system such as descending inhibition and/or diffuse noxious inhibitory controls (DNICs) in the brain stem [20-22]. Therefore, we think that acupuncture stimulation at the affected muscle may be most effective on the improvement in the pain thresholds.

The present study aims to determine whether acupuncture needling can relieve muscle pain. Effects of different depths of the needle penetration for relieving muscle pain under the DOMS model were also compared.

## Methods

### Participants

A total of 22 healthy volunteers (8 male and 14 female) aged between 18 and 28 years (mean 21.9 years) gave informed consent and participated in the study. All participants were recruited from the students of Meiji University of Integrative Medicine (Kyoto, Japan), in good health and not engaged in any physical training programs involving the exercise of the extensor digital muscle. At least six months after the first trial were required for a participant to be recruited again for the evaluation of the contra lateral side. This study was approved by the Ethics Committee of Meiji University of Integrative Medicine.

### Screening

All participants were screened for injury or pain (*eg *bone fracture, bruise and/or sprain of upper arm), medication, pregnancy, hemophilia, diabetes, asthma, weight-training, intense fear of needles and participation in any similar trial within the past year. All participants were instructed to avoid any form of exercise for the duration of the trial.

### Eccentric exercise

The participant sat on a chair with a movable weight (metal screw nut with a long-shaft bolt) attached to his/her third finger. The position of the weight (475 g) was adjusted such that the participant could remain the horizontal position for at least 10 seconds. The participant was asked to remain the position as long as possible. When bent 20 degrees downward at the matacarpophalangeal joint, the finger was reset to the original horizontal position manually by an investigator. This exercise was repeated until the participant was exhausted; three sets of the loaded exercises were performed with five minutes resting period. During the exercise, electromyogram (EMG) of the extensor digital muscle was monitored and displayed on an oscilloscope. Inappropriate movement by the participant during the exercise was corrected by the investigator when necessary.

### Randomization

A research assistant who was otherwise not involved in the study screened and enrolled participants at a research desk. After participants completed the eccentric exercise, another research assistant, who was not involved with data collection, randomly assigned them to one of the four treatment groups using a computer program (SAMPSIZE V2.0, Blackwell Science, USA), and blocked random-allocation sequence with a block size of four.

### Experimental conditions

#### Control group

Participants in this group rested supine on a standard treatment plinth for 30 minutes. The most tender point in the target muscle was treated.

#### Skin acupuncture at the tender point of ipsilateral muscle (skin group)

Participants in this group received needling at the maximum tender point typically located on the distal third of the belly of the extensor digital muscle. Disposable stainless steel needles (0.18 mm × 40 mm, Seirin, Japan) were inserted straight with a depth of 3 mm and retained in place for 30 minutes.

#### Muscle acupuncture at tender point of ipsilateral muscle (muscle group)

Participants in this group received needling on the maximum tender point which was the same as that in the skin group. Disposable stainless steel needles (0.18 mm × 40 mm, Seirin, Japan) were inserted straight with a depth of 10 mm and retained in place for 30 minutes.

#### Muscle acupuncture at tender point of non-segmental muscle (NS group)

Participants in this group received needling on the maximum tender point typically located on the distal third of the belly of the anterior tibial approximately over the musculotendinous junction. Disposable stainless steel needles (0.18 mm × 40 mm, Seirin, Japan) were inserted straight with a depth of 10 mm and retained in place for 30 minutes.

Acupuncture was performed by one of the authors (KI) who had three years of acupuncture training and ten years of clinical experience.

### Measurements

Pressure pain threshold (PPT) and electrical pain threshold (EPT) was measured at the point 20 mm distal to the maximum tender point. Pressure pain threshold (PPT) was determined as the minimum pressure (indicated as arbitrary units) that elicited the sensation of tenderness with a finger type pressure algometer (a probe of 6 mm in diameter) [15]. Measurement was repeated three times and the minimum value was employed as the threshold value.

Electrical pain thresholds (EPT) of skin, fascia and muscle were measured with a pulse algometer [15,16]. A stainless steel needle electrode insulated with acrylic resin (180 μ m in diameter, impedance 391 ± 30 kΩ at 1 kHz; Nisin Medical Institute, Japan) was used as a cathodal monopolar stimulating electrode. The needle was inserted manually and held in a guide tube attached to skin with adhesive tape. A metal surface anodal electrode was attached to the skin 10 mm apart from the needle. Participants were requested to press the button when he felt painful sensation (pain threshold), which triggered the digital display of the stimulus current and terminates the current stimulus pulse.

Needle was inserted stepwise at 0.5-1.0 mm and measured the pain thresholds of the skin, fascia and muscle. Depth in the fascia was determined by the needling stiffness (physical resistance for the manual insertion of needle) alongside with ultrasonic echo imaging (LOGIQ™400, GE Medical Systems, Japan).

Measurement was taken by one of the authors (YM) who had not been informed of the treatment allocation.

### Experimental schedule

This study was designed as an observer-blinded, randomized and controlled clinical trial. All participants were evaluated with PPT and EPTs. Participants then took part in the eccentric exercise and were allocated randomly to one of four groups after exercise. Two days following the exercise, all participants received treatment for approximately 30 minutes and were evaluated with PPT and EPTs immediately after treatment.

### Statistical analysis

PPT and EPT values were shown as mean ± standard deviation (SD). One-way analysis of variance (one-way ANOVA) followed by Dunnet's multiple comparison test (Statview for Windows, version 5.0, USA) were used to detect significant difference between groups in the EPT values. Multiple regression (Version 12, SYSTAT Software Inc., USA) was applied to analyzing the PPT values immediately after acupuncture stimulation (final PPT) between groups (control, skin, muscle and NS) with the following model: Final PPT = constant + baseline PPT +control+ skin + muscle + NS + error, where skin, muscle and NS were treated as dummy variables [20]. *P *< 0.05 was considered statistically significant.

## Results

### Changes in pressure pain threshold

Immediately after the repetitive eccentric exercise, the participants in all groups felt warmth and tenderness in the working muscle of the extensor digital muscle. Tenderness area was gradually restricted to the musculotendinous junction, and a rope-like taut band was detected in the tenderness area two days after the exercise.

**Figure 1:**
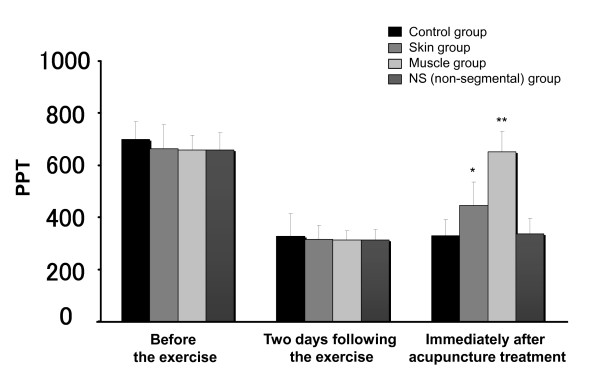
**Changes in pressure pain threshold (PPT) values after acupuncture**. Pressure pain threshold was measured with a finger type pressure algometer consisting of a strain gage and a processing unit. Changes in the resistance caused by strain in the gage was converted to changes in the voltage *via *Wheatstone bride in the processing unit which enables quantitative indication of the pressure as an arbitrary units (AU). A significant decrease in the PPT two days after exercise was found in all groups while recovery was observed after application of acupuncture at the skin and the muscle with statistical significance when compared with that in the control at the end of the trial. (*P *= 0.020 and *P *< 0.001 for skin and muscle respectively). Asterisks indicate significant differences compared with two days after the exercise (* *P *< 0.05, ** *P *< 0.001). Data are shown as mean ± SD.

While a significant decrease in the PPT values two days after the exercise was found in all groups, a statistically significant recovery was observed after acupuncture treatment at the skin and muscle (regression coefficients [estimated differences between controls and the rest of the groups] for the skin and the muscle were 111.3 [95%CI 19.5-203.0, *P *= 0.020] and 318.1 [95%CI 226.0-410.2, *P *< 0.001]) respectively (Figure 1, Table 1).

**Table 1:** Changes in electrical pain threshold (EPT) values after acupuncture

PPT (AU)	Control group (mean ± SD)	Skin group (mean ± SD)	Muscle group (mean ± SD)	Non-segmental group (mean ± SD)
**Before the exercise**	698.2 ± 68.6	663.0 ± 93.9	659.3 ± 56.9	658.8 ± 67.0
**Two days following the exercise**	327.0 ± 87.5	315.1 ± 54.1	313.8 ± 35.3	313.7 ± 39.3
**Immediately after acupuncture treatment**	328.7 ± 63.0	445.0 ± 89.6	652.3 ± 77.1	33.5 ± 60.6

### Changes in EPT values

**Figure 2:**
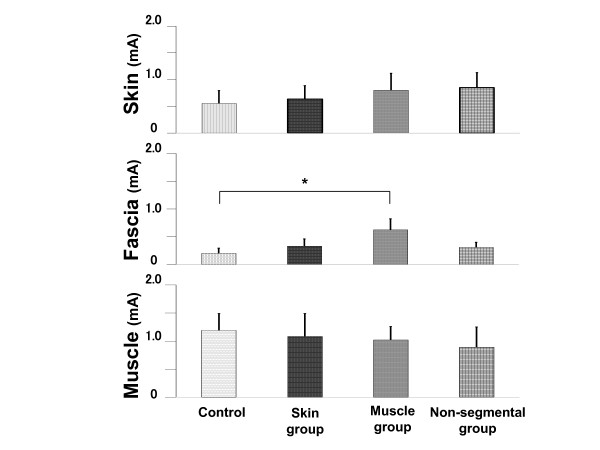
**Changes in electrical pain threshold (EPT) values after acupuncture**. Acupuncture to ipsilateral muscle (muscle group) only increased the EPT of fascia significantly (*P *= 0.028), but skin (skin group) and non-segmental muscle (non-segmental group) had no effect at all tissues (in terms of depths). Asterisk indicates significant differences compared with the control group (* *P *< 0.05). Data are shown as mean ± SD.

EPT values of the fascia of the muscle group in the second day were significantly higher than those in the control group (*P *= 0.028); however, no significant difference was found among the four groups in the EPT values of the skin and muscle groups (Figure 2, Table 2). Moreover, no significant difference was observed between the control and skin or NS group in the EPT values of all tissues (in terms of depths). EPT values of the fascia showed a similar pattern to that of the PPT values in Figure 1.

**Table 2:** Changes in electrical pain threshold (EPT) values after acupuncture

EPT (mA)	Control group (mean ± SD)	Skin group (mean ± SD)	Muscle group (mean ± SD)	Non-segmental group (mean ± SD)
**skin**	0.55 ± 0.64	0.64 ± 0.25	0.80 ± 0.32	0.85 ± 0.28
**fascia**	0.09 ± 0.12	0.33 ± 0.13	0.64 ± 0.20	0.31 ± 0.09
**muscle**	1.19 ± 0.30	1.09 ± 0.40	1.02 ± 0.24	0.89 ± 0.36

## Discussion

In the present study, two of the 22 participants recruited originally were asked to take part in the study again because the number of participants was not enough for four groups containing six participants each. A statistically significant difference was found only between the acupuncture stimulation of muscle (*P *< 0.001) and skin (*P *= 0.020) groups immediately after treatment, suggesting that acupuncture stimulation of muscle is effective for DOMS.

Several studies on the efficacy of acupuncture and/or dry needling treatment for pain conditions [12,23,24] indicated three important parameters, namely site, mode and intensity of the stimulation, are important for achieving efficacy of acupuncture and/or dry needling [12]. In past studies, the stimulation sites were traditional acupoints [25-27]; however, our recent studies suggested that the response to tenderness points, such as trigger points, could be greater than that to the traditional acupoints or non-tenderness points [14,28]. Moreover, our previous trial found that needling at the tender point on the affected muscle were more effective in the treatment of neck pain than that in the non-affected muscle [28]. The present study provided further evidence by demonstrating a statistically significant difference (*P *< 0.001) between the affected muscle (segmental muscle: the extensor digital muscle) and the non-affected muscle (non- segmental muscle: the anterior tibial muscle) on pressure pain thresholds, suggesting that tender point on the affected muscle may be more effective in treatment of muscle pain than the non-affected muscle.

Furthermore, the present study demonstrated a difference among the depths on pressure pain thresholds and electrical pain thresholds. As previous studies found that the applied pressure was transmitted to the muscle tissue through the skin and subcutaneous tissue, and activated muscle nociceptors responsible for pain sensation in the muscle [18,29,30], the thickness and physical properties of these tissues may strongly influence the pressure transmission. Moreover, the pressure obviously excited cutaneous receptors and probably induced pain sensation in the skin [30]. Thus, the origin of the pain was unclear in previous studies. On the other hand, electrical pain thresholds of skin, fascia and muscle were measured with a pulse algometer [15,16]. The pulse algometry used in the present study allowed us to measure deep pain threshold and selectively at different sites. Thus, the origin of the pain became clear in the present study.

Our results (Figure 1 and 2)showed that among the tested depths (5-10 mm) the acupuncture stimulation on the affected muscle at 10 mm was most effective on the improvement in the PPT and EPT. The present study was limited by its small sample size. Further studies of larger sample size are warranted.

## Conclusion

The present study shows that acupuncture stimulation of muscle increases the PPT and EPT of fascia. The depth of needle penetration is important for the relief of muscle pain.

## Abbreviations

EMG: electromyogram; PPT: pressure pain threshold; EPT: electrical pain threshold; NS: non-segmental; AU: arbitrary units; DOMS: delayed onset muscle soreness; DNICs: diffuse noxious inhibitory controls; SD: standard deviation.

## Competing interests

The authors declare that they have no competing interests.

## Authors' contributions

KI designed the study, performed the acupuncture treatment and wrote the manuscript. YM and HK designed and performed the statistical design and data analysis. All authors read and approved the final version of the manuscript.
